# Stuffing Teeth

**Published:** 1866-07

**Authors:** 


### STUFFING TEETH.

Editor Dental Register:—I was rejoiced to see an article in the editorial department, of the March No., on "stuffing teeth." There is a great reform needed in the operative department of our profession. Too many, who claim a high standing based upon the fact of their having maintained a long and lucrative practice, rest satisfied to jog along in the old beaten track, and continue to "stuff" teeth after the most "ancient and approved style," without ever giving a thought to the advancement of those much younger than themselves, and the rapid strides made by many around them. They represent themselves as the old reliable dentists, who are not so silly as to take up with every new fangled style of filling teeth, and do not require the aid of the "mallet," or any such "instruments of torture," or, as a gentlemau in the east has styled it, "a merciless tool in the hands of adventurers," (whom, by the way, I saw filling a tooth with it last summer, in Chicago.) These men need spurring up, they are daily doing incalculable injury to those who, with all confidence, place themselves under their care, thinking they are obtaining the highest skill; till by some lucky chance, or a dispensation of Divine Providence in their favor, these poor deluded patients come in contact with some one who has a proper appreciation of his profession, and is willing to base his standing upon the merits of his operations. They maintain their practice by keeping their patients in ignorance, and never allowing them, for a moment, to suspect that there are other dentists as good as themselves. Their patients are perfectly astonished when told that the decidious teeth should not be extracted till the permanent ones begin to appear—they have been instructed to have them extracted as soon as they begin to loosen or ache a little. They are incredulous when told that the six-year molars are the first permanent ones, and laugh at the very idea of treating and filling teeth at that age. I could relate a number of instances similar to the one you mentioned, but have occupied enough space already. If some of these gentlemen would only read the *Dental Register*, they might be made ashamed of their miserable practice, and their worse than miserable operations.

